# The Frailty, Fitness, and Psychophysical/Social Condition of Community-Dwelling Older Adults—Analysis of 5-Year Longitudinal Data

**DOI:** 10.3390/geriatrics10030082

**Published:** 2025-06-16

**Authors:** Emi Yamagata, Yuya Watanabe, Miwa Mitsuhashi, Hidemi Hashimoto, Yuriko Sugihara, Naoko Murata, Mitsuyo Komatsu, Naoyuki Ebine, Misaka Kimura

**Affiliations:** 1 Faculty of Nursing, Doshisha Women’s College of Liberal Arts, 97-1 Minamihokotate, Kodo, Kyotanabe 610-0395, Kyoto, Japan; mmitsuha@dwc.doshisha.ac.jp (M.M.); hihashim@dwc.doshisha.ac.jp (H.H.); ysugihar@dwc.doshisha.ac.jp (Y.S.); nmurata@dwc.doshisha.ac.jp (N.M.); mkomatsu@dwc.doshisha.ac.jp (M.K.); 2 Faculty of Sport Study, Biwako Seikei Sport College, 1204 Kitahira, Otsu 520-0503, Shiga, Japan; watanabe-yuy@bss.ac.jp; 3 Faculty of Health and Sports Science, Doshisha University, 1-3 Tatara Miyakodani, Kyotanabe 610-0394, Kyoto, Japan; nebine@mail.doshisha.ac.jp; 4 Institute for Active Health, Kyoto University of Advanced Science, 1-1 Nanjo Otani, Sogabe-cho, Kameoka 621-8555, Kyoto, Japan; kimura.misaka@kuas.ac.jp

**Keywords:** fitness age score, geriatric depression scale, Lubben social network scale short form, fitness testing event

## Abstract

**Background/Objectives:** Frailty is a multifactorial condition influenced by physical and psychosocial factors. Understanding longitudinal changes in these domains may guide prevention strategies. This study examines the relationship between frailty status, physical fitness, and psychosocial conditions in community-dwelling older adults using five-year longitudinal data. **Methods:** Participants were 52 out of 89 older adults who completed both baseline and five-year follow-up assessments (follow-up rate: 58.4%). Data were collected using 10 physical fitness indicators, the fitness age score (FAS), geriatric depression scale (GDS), Lubben social network scale short form (LSNS-6), and relevant items in the six Kihon Checklist (KCL) domains. Due to low prevalence of frailty, individuals with pre-frailty and frailty were combined into the frailty-risk group. Repeated measures ANOVA with sex as a covariate was conducted to compare groups. Logistic regression was used to identify baseline predictors of frailty status at five years. Statistical significance was set at *p* < 0.05. **Results:** GDS, LSNS-6, and KCL scores remained stable over five years. However, physical fitness significantly declined in several measures, including grip strength, vertical jump height, knee extension strength, functional reach, and FAS. A significant interaction for the timed up and go test showed that the robust group maintained function, while the frailty-risk group declined. Logistic regression identified KCL oral function as a significant predictor (OR = 5.331, 95% CI = 1.593–17.839, *p* = 0.007). **Conclusions:** Maintaining both oral function and physical fitness is vital for preventing frailty, even among health-conscious older adults. Proactive strategies may support healthy aging.

## 1. Introduction

Frailty, an age-associated decline in physiological reserve, is a major concern in Japan, a super-aging society. It increases vulnerability to stress, making one more prone to functional impairment and even death [1]. However, it is also considered a reversible condition. Therefore, appropriate interventions can prevent adverse health outcomes and facilitate one’s transition to robustness. The prevalence of frailty is approximately 10–20% among community-dwelling older adults, while the prevalence of pre-frailty is nearly 45–65% [2,3,4,5,6,7]. It is also important to note that the risk of frailty increases with advancing age [3,4,8]. Moreover, frailty is not a static condition but a dynamic one that can either improve, remain stable, or deteriorate over time [9,10,11,12,13,14,15,16]. In 2024, Japan’s aging rate reached a record high of 29.3%, comprising 12.5% early-stage older adults and 16.8% late-stage older adults [17]. This demographic shift highlights the urgent need for effective strategies to prevent and reverse frailty.

Meanwhile, research on factors associated with frailty has been steadily accumulating. Longitudinal studies with follow-up periods ranging from several years to 12 years have reported associations between frailty and various factors, including physical activity, subjective awareness of lower limb muscle weakness, energy intake, dietary quality, oral function, metabolic syndrome, cognitive function, depression, and social engagement [9,12,13,18,19,20,21,22].

In addition, physical fitness, a multi-component physical health indicator, is also reportedly associated with frailty. Navarrete-Villanueva et al. [23] demonstrated that among various physical fitness components, gait speed, aerobic capacity, lower limb strength, and grip strength are particularly associated with frailty.

However, few studies simultaneously assess physical aspects—including multiple components of physical fitness—and psychosocial factors for more than 5 years. To advance understanding in this area, it is imperative to accumulate evidence through mid- to long-term longitudinal studies incorporating multifactorial assessments. Therefore, this study aimed to investigate the relationship between frailty status, multiple components of physical fitness, and psychosocial conditions over a five-year period among community-dwelling older adults. Through this investigation, we aim to identify which of these factors are associated with frailty risk after five years, contributing to improved prevention strategies.

## 2. Materials and Methods

### 2.1. Study Design

This longitudinal study was part of an annual fitness testing event targeting individuals aged 40 years or older living near City A. The baseline surveys were conducted in June 2016 and March 2017, and participants were recruited through public announcements in the city’s public relations magazine and local newspapers. For subsequent surveys, invitations were mailed to individuals who had participated in previous years. The survey conducted in March 2022 was designated as the five-year follow-up assessment.

In addition to physical fitness indicators, the testing items were designed to comprehensively capture factors associated with frailty, including physical, psychological, and social components, based on the integral conceptual model of frailty [24]. The same researcher was responsible for organizing the fitness testing event and conducting each primary measurement throughout the five-year period, while assistant examiners changed each year.

### 2.2. Participants

**Figure 1:**
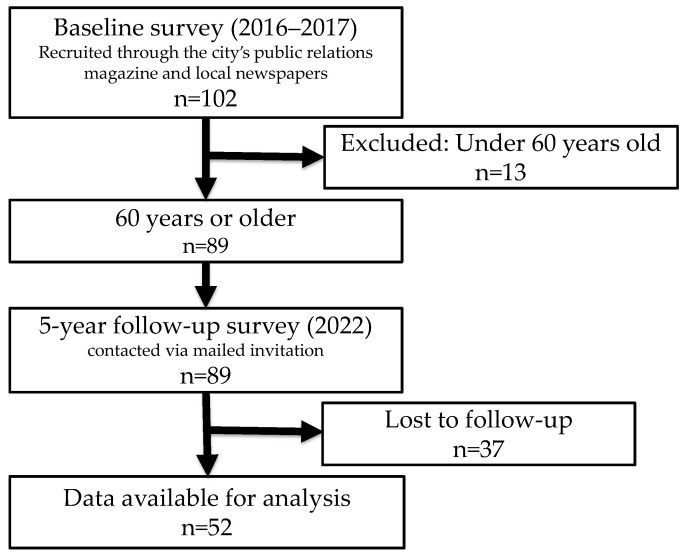
Flow chart for inclusion and exclusion of research subjects.

This study focused on individuals aged 65 or older at the time of the follow-up survey (i.e., those aged 60 or older at baseline survey). Of the 102 individuals who participated in the baseline survey, 89 were 60 years old or older. Data from 52 of these individuals (58.4%) who participated in the follow-up survey were analyzed (Figure 1).

### 2.3. Measurements

#### 2.3.1. Physique and Physical Fitness

Height and weight were measured as indicators of physique, and body mass index (BMI) was calculated by dividing the weight by the square of the height. Physical fitness was evaluated based on 10 items: grip strength, knee extension strength (KES), 30 s chair standing frequency, vertical jump height, chair stepping, single-leg stance with eyes open, sit-and-reach, functional reach (FR), 10 m walking speed, and timed up and go (TUG). Table 1 presents a summary of each measurement protocol. All measurements were conducted following established procedures described in previous studies [25,26,27].

The physical fitness instruments used in this study were manufactured in Japan and have been widely utilized in prior studies involving Japanese older adults. Specifically, the devices were produced by Takei Scientific Instruments (Niigata, Japan) and Panasonic (Osaka, Japan), both of which are widely used in domestic geriatric research and practice. Although not all tools have been formally validated in cross-cultural studies, they are commonly accepted for use in physical fitness assessments among older adults in Japan. In all physical assessments, brief rest periods were incorporated into the protocol and adjusted according to each participant’s pace.

In addition, we calculated the fitness age score (FAS), a comprehensive indicator of physical fitness [25,26,28]. The FAS calculation involved five items that reflect age-related changes: 10 m walking time, functional reach, single-leg stance with eyes open, vertical jump height, and grip strength. The FAS formulas were as follows:

FAS among men = −0.203 X1 + 0.034 X2 + 0.0064 X3 + 0.044 X4 + 0.046 X5 − 3.05

FAS among women = −0.263 X1 + 0.033 X2 + 0.0074 X3 + 0.048 X4 + 0.079 X5 − 2.52

where X1 is 10 m walking time (normal walking) in seconds, X2 is functional reach (FR) in centimeters, X3 is single-leg stance with eyes open in seconds, X4 is vertical jump height in centimeters, and X5 is grip strength in kilograms.

#### 2.3.2. Basic Characteristics, Health Perceptions, and Lifestyle Factors

We collected data on age, sex, household composition, perceived health, presence of hobbies, role in the household, work with income (work), and participation in community activities. Household composition was assessed using the following seven response options: “living alone”, “living with spouse only”, “living with children”, “living with children and grandchildren”, “living with parents”, “living with parents and children”, and “other”. For cases such as “living with spouse and children,” which were not explicitly listed among the response options, participants were instructed to select “other”. For analysis, responses were classified into three categories: “living alone”, “living with spouse only”, and “other”. The “other” category included all other cohabitation types except for living with spouse only. Participants were asked to select a single option that best reflected their current living situation. Health perception was measured using a 4-point Likert-type scale with the following response options: “very healthy”, “somewhat healthy”, “somewhat unhealthy”, and “very unhealthy”. Participants were instructed to select the option that best reflected their current perception of their health. We categorized the first two options as the healthy group and the last two as the unhealthy group. Participation in community activities, including volunteer work and local activities, was assessed using three response options: “participate frequently”, “participate sometimes”, and “do not participate”. We categorized the first two options as the participation group and the last option as the non-participation group. Other items were answered with “yes” or “no”.

#### 2.3.3. Frailty

Frailty was assessed based on the Japanese version of the cardiovascular health study (J-CHS) criteria [29], according to which frailty is evaluated using five items: weight loss (loss of 2–3 kg or more in 6 months), reduced vitality (feeling inexplicably fatigued in the past two weeks), decreased physical activity (not engaging in light exercise or regular exercise/sports), grip strength (less than 28 kg for men, less than 18 kg for women), and walking speed (less than 1.0 m/second). The fulfillment of three or more criteria, one or two criteria, and no criteria denoted the presence of frailty, pre-frailty, and robustness, respectively. Grip strength and walking speed were assessed using the abovementioned methods, while weight loss, reduced vitality, and decreased physical activity were evaluated using a questionnaire.

#### 2.3.4. Depression

Depression was assessed using the geriatric depression scale (GDS) [30,31], which comprises 15 questions answered with “yes” or “no.” The maximum score on the scale is 15, and higher scores suggest depression.

#### 2.3.5. Social Isolation

Social isolation was assessed using the Japanese version of the Lubben social network scale short form (LSNS-6) [32]. This scale comprises six questions related to family and non-family networks, with questions answered on a six-point scale. The maximum score is 30; higher scores indicate more robust social networks.

#### 2.3.6. The Kihon Checklist (KCL)

KCL comprises 25 questions across seven domains: instrumental activities of daily living (IADL), physical function, nutritional status, oral function, homebound state, cognitive function, and mood [33,34,35,36]. We calculated scores on the complete checklist (total score) and the score for seven domains.

### 2.4. Data Analysis

We determined the number of individuals with frailty, pre-frailty, and robustness in the baseline and follow-up surveys. The difference in the prevalence rates between the two surveys was compared using chi-squared tests. Because only one participant was classified as frail at follow-up, we combined participants with frailty and pre-frailty into a single “frailty risk” group for analysis. In contrast, those classified as robust were categorized as the “robust group”. Age and sex distributions between the robust and frailty risk groups at follow-up were compared using independent *t*-tests and chi-squared tests. Group comparisons of health perceptions and lifestyle factors in each survey were also conducted using chi-squared tests. To investigate physique, physical fitness, depression, social isolation, and KCL in the robust and frailty risk groups, we performed a factorial repeated measures analysis of variance (ANOVA, Group × Time) with sex as a covariate. Bonferroni’s multiple comparison tests were applied in the case of significant interactions. A priori sample size calculation was conducted using G*Power (version 3.1). Assuming an effect size of f = 0.4, an alpha level of 0.05, and a statistical power of 0.80, a repeated measures ANOVA with two groups and two time points required a total of 52 participants. The sample size used in this study (*n* = 52) was therefore deemed sufficient for the planned analyses.

In addition, logistic regression analysis was conducted with frailty status at 5 years as the dependent variable and gender, interacting items, and items showing significant differences between groups in the baseline survey as independent variables. To assess multicollinearity in the logistic regression model, Spearman’s rank correlation coefficient was used to analyze the correlation between the independent variables. The correlation coefficients were confirmed to be below 0.9. Additionally, the variance inflation factor (VIF) was calculated for the variables, and all VIF values were confirmed to be below 10 [37]. To confirm the goodness-of-fit of the model, the omnibus test of the model coefficients and the Hosmer–Lemeshow goodness-of-fit test results were examined. These statistical analyses were performed using IBM SPSS Statistics version 29 (IBM Japan, Tokyo, Japan). For all statistical tests, *p* < 0.05 was considered significant.

## 3. Results

### 3.1. Characteristics of the Participants

Of the 52 participants included in the study, 20 (38.5%) were men and 32 (61.5%) were women. At baseline, the mean age of participants was 72.5 ± 4.6 years (range: 62–84 years). Table 2 shows frailty status at the baseline and follow-up surveys in this study. Among 29 robust participants at baseline, 19 (65.5%) remained robust at the follow-up. Among 23 pre-frail participants, 4 (17.4%) became robust, and 18 (78.3%) remained pre-frail.

For analytical purposes, participants who were categorized as either pre-frail or frail at follow-up were combined into a single group, referred to as the “frailty risk group”.

Table 3 compares the mean age and sex distribution in the robust and frailty risk groups at follow-up. The robust group comprised 23 robust participants, while the frailty risk group comprised 29 participants with frailty or pre-frailty. There were no significant differences in the mean age or sex distribution between the two groups.

### 3.2. Health Perception and Lifestyle Factors in the Robust and Frailty Risk Groups

Table 4 presents a comparison of health perceptions and lifestyle factors between the robust and frailty risk groups at baseline and follow-up. A significant difference was found only at follow-up, where the proportion of participants with paid work was significantly lower in the frailty risk group compared to the robust group (*p* = 0.044).

### 3.3. Physique, Physical Fitness, and Psychosocial Conditions in the Robust and Frailty Risk Groups

Table 5 summarizes the comparison of physique, physical fitness, and psychosocial conditions between the robust and frailty risk groups at baseline and follow-up. At baseline, the robust group showed significantly higher values in grip strength, vertical jump height, the 30 s chair stand test, and FAS (*p* < 0.05). A significant interaction was found for TUG (*p* = 0.011): post hoc analysis indicated that the frailty risk group showed a significant increase over time (*p* < 0.001), while no significant change was observed in the robust group. No other significant interactions were observed, though trends were noted for single-leg stance with eyes open (*p* = 0.081) and KES (*p* = 0.087). Height, grip strength, vertical jump height, KES, FR, and FAS exhibited significant time effects, all showing declines over the five-year period (*p* < 0.05).

Regarding psychosocial conditions, GDS and LSNS-6 scores changed slightly over time in both groups, but no significant group differences, interactions, or time effects were found. For the KCL, baseline differences were significant in oral function (*p* = 0.002) and homebound status (*p* = 0.022), with the frailty risk group scoring higher. Although repeated measures ANOVA showed no significant interactions, a trend was observed in the total KCL score (*p* = 0.050).

### 3.4. Factors Associated with Frailty Risk After 5 Years

The independent variables in the logistic regression analysis included eight variables identified in previous analyses: sex, grip strength, 30 s chair standing test, vertical jump height, TUG, FAS, KCL oral function, and KCL homebound. However, the KCL homebound variable exhibited complete separation with the dependent variable, resulting in unstable coefficient estimates. Therefore, this variable was excluded from the model to ensure analysis stability. The results showed statistical significance in the Omnibus test of model coefficients (*p* < 0.001), and the Hosmer–Lemeshow goodness-of-fit test yielded a *p*-value of 0.147, suggesting a good fit for the model. The analysis revealed a significant association with KCL oral function (odds ratio [95% confidence interval] = 5.331 [1.593–17.839], *p* = 0.007) (Table 6).

## 4. Discussion

This study investigated the relationship between older adults’ frailty status, physical fitness, and psychosocial conditions using five-year longitudinal data. At baseline, 65.5% of robust participants remained robust five years later. In contrast, 82.6% of those classified as pre-frail at baseline either maintained their pre-frail state or transitioned to frailty. Regarding the psychosocial conditions, no significant changes were observed in GDS, LSNS-6, and KCL scores over the 5 years. However, physical fitness significantly decreased in several measures, including grip strength, vertical jump height, KES, FR, and FAS. The comparison between the robust and frailty risk groups revealed a significant interaction effect for the TUG, indicating that the robust group maintained their function. In contrast, the frailty risk group experienced a significant decline. Furthermore, higher baseline scores on KCL oral function were identified as a significant predictor of being classified as pre-frail or frail five years later.

Previous studies have reported that a proportion of individuals with frailty or pre-frailty improve their frailty status over time [12,13]. For example, Oishi et al. [14] conducted a five-year longitudinal study of 124 older Japanese adults and reported that 45% maintained a robust status, while 74.5% of those who were pre-frail remained pre-frail or progressed to frailty after five years. Others reported that 40.5–76.0% of robust individuals maintained their robustness over a one- to two-year period [10,11,12,13,16], while 41.5–75.0% of pre-frail or frail individuals maintained the same state [10,11,12,13,15,16]. These previous studies used diverse and unstandardized frailty assessment indicators and had varying follow-up periods, making direct comparisons difficult. Nevertheless, in this study, both the proportions of individuals who maintained a robust status over five years (65.5%) and those who either remained in a pre-frail state or progressed from pre-frailty to frailty (82.6%) were slightly higher than those reported by Oishi et al. [14].

Our findings indicate that, even among community-dwelling older adults who regularly participate in fitness testing events, a certain proportion did not show improvement from pre-frailty but, instead, remained pre-frail or progressed to frailty. These results underscore the necessity of implementing frailty prevention measures even for this relatively health-conscious population.

Previous studies have reported GDS scores ranging from 3.3 to 5.0 and LSNS-6 scores ranging from 13.0 to 22.9 among community-dwelling older adults in Japan [38,39,40,41,42]. Depression is considered a psychological aspect of frailty, while social isolation represents its social aspect. In this study, GDS scores ranged from 2 to 3 points, and LSNS-6 scores ranged from 16 to 20 points in both groups and across both surveys (Table 5). These findings suggest that the participants in this study maintained their psychological and social conditions over the five years of study. Even among participants who voluntarily attended fitness assessments, a significant decline in physical fitness was observed over the five-year period. Among the various physical components evaluated, a significant interaction was observed for TUG performance in this study, and notable declines were also found in muscle strength (grip strength, KES), muscle power (vertical jump height), and balance (FR). TUG is a widely used measure of functional mobility and is closely associated with the ability to perform daily activities and to safely go out alone [43]. These capacities are fundamental to independent living, and interventions that promote their maintenance or improvement are therefore likely to be highly beneficial.

Repeated measures ANOVA indicated a significant group × time interaction for TUG performance, suggesting greater functional decline in the frailty-risk group. However, TUG was not identified as a significant predictor in the logistic regression model. This discrepancy likely reflects the difference in analytic focus: ANOVA assesses group-level changes over time, whereas logistic regression examines the independent contribution of each baseline variable to frailty risk at the individual level. It is also possible that the predictive effect of TUG was attenuated due to its overlap with other physical fitness indicators included in the model.

In addition, our results showed that the proportion of participants with paid work at follow-up was significantly lower in the frailty risk group than in the robust group (*p* = 0.044). Although this difference was statistically significant, it should be interpreted with caution given its proximity to the conventional significance threshold. Further research is needed to clarify the potential association. This finding raises the possibility that employment status may be related to the observed decline in physical fitness. However, previous studies have reported conflicting findings: one study indicated that physical capacity is not necessarily associated with work ability in older adults [44], while another found that retirement may be linked to increased physical activity [45]. These inconsistencies highlight the need for further investigation into the complex relationship between work status and physical function in later life.

Nagai et al. [46] reported that reduced walking speed and decreased muscle strength are significant risk factors for social frailty. In line with their findings, the present results suggest that a decline in physical fitness may precede changes in psychological and social conditions, supporting the notion that early physical deterioration can be an initial indicator of frailty. These observations align with previous findings and reinforce the importance of monitoring physical function, not only for assessing current status but also for anticipating future psychosocial decline.

Therefore, regular physical fitness assessments may be an effective means of detecting functional decline and potential frailty early. Participation in fitness testing events may also offer additional benefits, including increased awareness of physical condition, self-monitoring of health and functional capacity, and support for planning future health maintenance strategies [47]. The implementation of such events—even on an annual basis—may enhance older adults’ motivation to maintain their health and contribute to ongoing improvements in frailty prevention and overall well-being.

The present findings reveal that higher KCL oral function significantly influenced the incidence of frailty and pre-frailty five years later. Previous studies [48,49,50] have shown that oral function and oral frailty are predictors of physical frailty, adverse health outcomes, and mortality. Our results support these earlier findings. Furthermore, among the various physical, psychological, and social factors examined in this study, KCL oral function emerged as a particularly influential factor. This suggests the necessity of providing support for maintaining and improving oral function, even among individuals who are motivated to participate in fitness testing events. Oral function is linked not only to nutritional intake but also to social interaction and communication ability [51], which are known to contribute to both physical and psychological resilience in older adults. Early detection of declining oral function through regular assessments, as well as community-wide awareness-raising activities aimed at maintaining oral health in older adults, may be important strategies for frailty prevention.

### Strengths and Limitations

There were several limitations to this study. First, the sample size was limited (*n* = 52), and the participants could not be considered representative of the older population in City A. This limits the generalizability of the findings to the broader community-dwelling older adult population. Although logistic regression was performed with six predictor variables, the sample size did not meet the commonly recommended minimum (i.e., 10 cases per variable), potentially affecting the stability and reliability of the model. The small sample may also have limited the statistical power to detect moderate associations, particularly in multivariable and interaction analyses.

Second, the participants were individuals who regularly attended fitness testing events; those who discontinued or never participated were not included. These participants may have had higher health awareness and motivation compared to the general older adult population, which could have influenced the study outcomes. In addition, among the 89 participants at baseline, the background characteristics of the 37 individuals who did not participate in the five-year follow-up survey were not tracked. The lack of information on these individuals introduces potential attrition bias, as their characteristics may differ systematically from those who completed the study.

Third, data on participants’ physical activity levels and related behaviors during the follow-up period were not collected, making it difficult to fully evaluate the observed changes in physical fitness. Notably, the follow-up survey was conducted in March 2022, after the COVID-19 outbreak. The pandemic may have influenced participants’ physical fitness and psychosocial conditions; however, the extent of this impact remains unclear.

Fourth, due to the limited sample size and the absence of significant differences in sex distribution between the robust and frail groups, we did not perform stratified or three-way interaction analyses. However, we incorporated sex as an independent variable in the logistic regression model to account for potential sex-related effects in a statistically robust manner. Previous studies have reported sex differences in frailty prevalence and progression, and future studies with larger samples should incorporate comprehensive sex-based analyses to inform more tailored prevention strategies.

Despite these limitations, this study provides valuable insights. Few longitudinal studies have examined transitions to frailty from a multidimensional perspective, including physical fitness. The findings offer important information for developing effective strategies to prevent frailty. Furthermore, the results highlight the need for frailty prevention even among relatively robust and health-conscious older adults who actively participate in fitness assessments. Interventions aimed at maintaining and improving physical fitness remain essential for promoting long-term health and well-being in this proactive population.

## 5. Conclusions

Even among health-conscious older adults who regularly participate in fitness testing events, it becomes increasingly difficult with age to maintain a robust status or improve from frailty. Maintaining both physical fitness and oral function is essential for preventing frailty. Supporting early-stage frailty prevention through opportunities such as fitness testing events may help promote healthy aging in the community.

## Figures and Tables

**Table 1:** Measurement protocols for physical fitness assessment items.

	Measurement Item (Unit)	Purpose	Device Used	Procedure Summary
1	Grip strength (kg)	Muscle strength	Digital grip strength meter (TKK5401: Takei Scientific Instruments, Niigata, Japan)	Each hand was tested twice with maximum effort. The higher value from each hand was averaged.
2	KES (kg)	Muscle strength	Leg strength measurement platform and tension meter (TKK5715, TKK5710e: Takei Scientific Instruments, Niigata, Japan)	Each leg was tested twice with maximum effort. The higher value from each leg was averaged.
3	30 s chair standing test (numbers)	Lower limb muscular function	Standard chair	One trial: number of chair stands completed in 30 s
4	Vertical jump height (cm)	Muscle power	Jump meter (TKK5406: Takei Scientific Instruments, Niigata, Japan)	Two maximal jumps with arm swing; the highest value was used
5	Chair stepping (numbers)	Foot movement agility	Chair and floor markers (30 cm)	Open/close feet while seated between floor lines (30 cm apart); each performed one trial; 20-sec count
6	Single leg stance with eyes open (seconds)	Balance and stability	Stopwatch	Two trials with eyes open; the longest one-leg stance time was recorded
7	Sit-and-reach (cm)	Flexibility	Digital flexibility tester (TKK5112: Takei Scientific Instruments, Niigata, Japan)	Sit-and-reach test with straight legs; each performed two trials; the greater distance was used
8	FR (cm)	Balance	Digital mirror (PN-S3019030: Panasonic, Osaka, Japan)	Reach forward from standing posture; each performed two trials; the max distance was used
9	10 m walking speed (m/s)	Gait speed	Stopwatch	10 m walk at their usual pace; each performed two trials; the average time was used to calculate speed
10	TUG (seconds)	Functional mobility	Digital mirror (PN-S3019030: Panasonic, Osaka, Japan)	Each performed one trial: stand from chair, walk 3 m and back, sit down; the total time was used

**Table 2:** Frailty status at the baseline and follow-up surveys.

		Follow-Up Survey						
		Robust Group		Frailty Risk Group				
		Robust		Pre-Frail		Frail		
		*n*	%	*n*	%	*n*	%	*p*
Baseline	Robust	19	65.5%	10	34.5%	0	0.0%	0.002
	Pre-frail	4	17.4%	18	78.3%	1	4.3%	
	Frail	0	0.0%	0	0.0%	0	0.0%	

Chi-square tests.

**Table 3:** Age and sex distribution in the robust and frailty risk groups at follow-up.

		Robust Group		Frailty Risk Group		*p*
Age (y) ^1^		77.4 ± 4.0		77.8 ± 5.3		0.745
Sex ^2^	Men	11	55.0%	9	45.0%	0.260
	Women	12	37.5%	20	62.5%	

^1^ Age (mean ± SD): Independent *t*-test. ^2^ Sex (*n*, %): Chi-square tests.

**Table 4:** Group comparisons of health perceptions and lifestyle factors in baseline and follow-up surveys.

			Robust Group		Frailty Risk Group		
			*n*	%	*n*	%	*p*
Baseline	Household composition	Living alone	5	21.7%	3	10.3%	0.526
		Living with spouse only	14	60.9%	20	69.0%	
		Other	4	17.4%	6	20.7%	
	Perceived health	Healthy	23	100.0%	27	93.1%	0.497
		Unhealthy	0	0.0%	2	6.9%	
	Presence of hobbies	Yes	23	100.0%	26	89.7%	0.245
		No	0	0.0%	3	10.3%	
	Role in the household	Yes	20	87.0%	26	89.7%	1.000
		No	3	13.0%	3	10.3%	
	Work with income	Yes	7	30.4%	5	17.2%	0.329
		No	16	69.6%	24	82.8%	
	Participation in community activities	Yes	6	26.1%	6	20.7%	0.746
		No	17	73.9%	23	79.3%	
Follow-up survey	Household composition	Living alone	6	26.1%	6	20.7%	0.206
		Living with spouse only	14	60.9%	13	44.8%	
		Other	3	13.0%	10	34.5%	
	Perceived health	Healthy	23	100.0%	25	86.2%	0.120
		Unhealthy	0	0.0%	4	13.8%	
	Presence of hobbies	Yes	22	95.7%	29	100.0%	0.442
		No	1	4.3%	0	0.0%	
	Role in the household	Yes	21	91.3%	29	100.0%	0.191
		No	2	8.7%	0	0.0%	
	Work with income	Yes	8	34.8%	3	10.3%	0.044
		No	15	65.2%	26	89.7%	
	Participation in community activities	Yes	8	34.8%	6	20.7%	0.348
		No	15	65.2%	23	79.3%	

Chi-square tests.

**Table 5:** The physique, physical fitness, and psychosocial conditions of the groups at the baseline and follow-up surveys.

	Robust Group						Frailty Risk Group									
	Baseline			Follow-Up			Baseline			Follow-Up			Time Effect		Interaction	
	Mean		SD	Mean		SD	Mean		SD	Mean		SD	F	*p*	F	*p*
Physique and physical fitness																
Height (cm)	159.7	±	8.7	159.1	±	8.8	156.6	±	6.8	155.6	±	6.7	7.170	0.010	3.419	0.070
Weight (kg)	56.5	±	10.7	57.0	±	11.4	56.5	±	8.1	56.0	±	7.8	1.428	0.238	1.565	0.217
BMI (kg/m^2^)	22.0	±	2.8	22.3	±	3.0	23.0	±	2.7	23.1	±	3.1	0.429	0.515	0.511	0.478
Grip strength (kg)	30.6	±	7.1 *	29.0	±	6.6	25.6	±	6.7	23.6	±	6.3	21.094	<0.001	1.587	0.214
KES (kg)	35.2	±	10.6	34.6	±	8.7	28.8	±	9.4	25.7	±	10.4	5.224	0.027	3.056	0.087
30 s chair standing test (numbers)	28.1	±	6.0 *	24.4	±	5.5	24.4	±	5.8	21.9	±	4.6	2.632	0.111	0.527	0.472
Vertical jump height (cm)	30.1	±	6.8 *	25.5	±	5.8	24.9	±	6.5	20.2	±	7.0	24.673	<0.001	0.348	0.558
Chair stepping test (numbers)	31.9	±	5.1	34.3	±	5.5	31.0	±	4.2	32.6	±	5.9	1.655	0.204	0.183	0.671
Single-leg stance with eyes open (s)	54.9	±	44.3	40.1	±	30.9	35.8	±	35.2	39.4	±	39.3	0.314	0.578	3.167	0.081
Sit-and-reach (cm)	36.1	±	13.9	32.6	±	12.5	39.2	±	8.6	33.9	±	10.9	1.706	0.197	0.066	0.798
FR (cm)	34.0	±	4.7	33.4	±	5.3	32.7	±	3.6	30.4	±	6.6	4.865	0.032	2.191	0.145
10 m walking speed (m/s)	1.43	±	0.2	1.46	±	0.2	1.37	±	0.1	1.43	±	0.2	0.000	0.991	0.194	0.661
TUG (s)	5.61	±	0.9	5.74	±	0.9	5.95	±	0.9	6.64	±	1.3 ^†††^	3.466	0.069	6.926	0.011
FAS	0.25	±	0.9 *	−0.10	±	0.7	−0.29	±	0.6	−0.62	±	0.8	8.241	0.006	0.020	0.889
Psychosocial conditions																
GDS	2.5	±	3.1	2.2	±	3.1	3.0	±	2.4	3.4	±	2.6	0.764	0.386	1.412	0.241
LSNS−6	18.3	±	6.1	19.3	±	7.0	16.6	±	4.3	17.0	±	4.5	0.116	0.735	0.320	0.575
KCL total score	2.78	±	2.4	2.52	±	1.8	3.97	±	2.8	5.21	±	3.1	0.034	0.855	4.045	0.050
KCL IADL	0.35	±	0.7	0.17	±	0.4	0.24	±	0.5	0.28	±	0.7	1.366	0.248	2.151	0.149
KCL physical function	0.96	±	1.1	0.70	±	0.8	1.00	±	1.0	1.38	±	0.9	0.790	0.378	4.005	0.051
KCL nutrition	0.22	±	0.4	0.04	±	0.2	0.38	±	0.6	0.48	±	0.6	1.613	0.210	3.605	0.063
KCL oral function	0.35	±	0.5 **	0.52	±	0.7	0.93	±	0.8	0.72	±	0.9	0.005	0.946	3.690	0.061
KCL homebound	0.04	±	0.2 *	0.26	±	0.5	0.28	±	0.5	0.52	±	0.5	0.004	0.950	0.000	0.991
KCL cognitive function	0.64	±	0.7	0.45	±	0.7	0.38	±	0.6	0.55	±	0.8	1.407	0.241	1.483	0.229
KCL depression	0.26	±	0.9	0.35	±	0.8	0.76	±	1.0	1.28	±	1.1	0.343	0.561	1.329	0.254

Covariate: sex; * *p* < 0.05, ** *p* < 0.01 group comparison at baseline; ^†††^ *p* < 0.001 comparison with baseline in each group.

**Table 6:** Logistic regression analysis of factors associated with frailty risk after 5 years.

	OR	95% CI	*p*
Sex ^1^	7.792	0.052–1163.22	0.421
Grip Strength	1.001	0.715–1.40	0.997
30 s chair standing test	0.866	0.733–1.02	0.093
Vertical jump height	0.965	0.785–1.19	0.737
TUG	0.855	0.221–3.30	0.821
FAS	0.254	0.019–3.42	0.302
KCL oral function	5.331	1.593–17.84	0.007

OR: odds ratio; 95% CI: 95% confidence interval. Dependent variable: robust group = 0, frailty risk group = 1. ^1^ Sex: men = 0, women = 1.

## Data Availability

The data that support the findings of this study are available from the corresponding author upon reasonable request.
